# Extracellular Vesicles in Acute Leukemia: A Mesmerizing Journey With a Focus on Transferred microRNAs

**DOI:** 10.3389/fcell.2021.766371

**Published:** 2021-10-06

**Authors:** Mehrdad Izadirad, Zoufang Huang, Farideh Jafari, Amir Ali Hamidieh, Ahmad Gharehbaghian, Yi-Dong Li, Leila Jafari, Zhe-Sheng Chen

**Affiliations:** ^1^Department of Hematology and Blood Bank, School of Allied Medical Sciences, Shahid Beheshti University of Medical Sciences, Tehran, Iran; ^2^Department of Hematology, The First Affiliated Hospital of Gannan Medical University, Ganzhou, China; ^3^Pediatric Cell and Gene Therapy Research Center, Gene, Cell and Tissue Research Institute, Tehran University of Medical Sciences, Tehran, Iran; ^4^Department of Pharmaceutical Sciences, College of Pharmacy and Health Sciences, Queens, NY, United States; ^5^Institute for Biotechnology, St. John’s University, Queens, NY, United States

**Keywords:** acute myeloblastic leukemia, acute lymphoblastic leukemia, extracellular vesicles, prognosis factor, disease pathogenesis, miRNAs, non-coding RNAs

## Abstract

Despite their small size, the membrane-bound particles named extracellular vesicles (EVs) seem to play an enormous role in the pathogenesis of acute leukemia. From oncogenic hematopoietic stem cells (HSCs) to become leukemic cells to alter the architecture of bone marrow (BM) microenvironment, EVs are critical components of leukemia development. As a carrier of essential molecules, especially a group of small non-coding RNAs known as miRNA, recently, EVs have attracted tremendous attention as a prognostic factor. Given the importance of miRNAs in the early stages of leukemogenesis and also their critical parts in the development of drug-resistant phenotype, it seems that the importance of EVs in the development of leukemia is more than what is expected. To be familiar with the clinical value of leukemia-derived EVs, this review aimed to briefly shed light on the biology of EVs and to discuss the role of EV-derived miRNAs in the development of acute myeloid leukemia and acute lymphoblastic leukemia. By elaborating the advances and challenges concerning the isolation of EVs, we discuss whether EVs could have a prognostic value in the clinical setting for leukemia.

## Introduction

As the most common type of leukemia, the incidence of acute leukemia, either acute myeloid leukemia (AML) or acute lymphoblastic leukemia (ALL), is increased in the last few years (Hunger and Mullighan, 2015; Park et al., 2015; Terwilliger and Abdul-Hay, 2017; Bosshard et al., 2018; Wiese and Daver, 2018). The aggressive and heterogeneous behavior of acute leukemia is originated from not only the diversity of genetic abnormalities but also the occurrence of extensive epigenetic changes. The list of involved genes and molecules in the pathogenesis of these malignancies is endless and new candidates are continuously emerging. This heterogenic characteristic of acute leukemia brings obstacles in the way of precise treatment of the disease and somehow ends the era of the conventional chemotherapeutic approaches (Bassan and Hoelzer, 2011; Selim and Moore, 2018). Despite the efficacy of the common chemotherapy regimen consist of cytarabine and anthracycline, which thus far is the most effective treatment strategy for leukemia patients, a considerable proportion of patients succumb to the disease due to the therapy failure (Bassan and Hoelzer, 2011; Luger, 2017).

Recently, a group of extracellular vesicles (EVs) derived from the cancer cells that carry the property of their parental cells ranging from signaling proteins to nucleic acids such as DNAs was successfully identified (Voso et al., 2019). It became evident that cancer cell-derived EVs are auxiliary tools that support cancer growth by carrying the factors that could enhance angiogenesis (Skog et al., 2008), provide the metabolic needs of tumor cells and promote their proliferative capacity (Fong et al., 2015). In hematologic malignancies, for example in leukemia, EVs were showed to participate in the primary tumor growth as well as inducing multi-drug resistance through recruiting microenvironment resident cells such as endothelial cells (ECs) or leukocytes (Litwińska et al., 2019). In ALL, it has been proposed that the communication between leukemic cells and the bone marrow residential cells could increase the survival of leukemic cells through exporting exo-RNAs. In pre-B ALL, for example, some evidence revealed that the delivered exo-miRNAs could change the architecture of the BM environment in the manner that it reinforces the proliferation of leukemic cells and suppresses the activity of immune system (Xu et al., 2018). MicroRNAs are one of the main non-coding RNAs that play a critical role in the development of leukemia. There are multiple evidence reporting that miRNA transferred by EVs into the leukemic cells could be fundamental in the pathogenesis of leukemia. For example, the transferred miR-155 to AML cells could suppress the expression of C/EBPA to reinforce the development of leukemogenesis (Alemdehy et al., 2016). Or, the upregulation of long non-coding RNA SNHG1 expression has also been suggested to elevate the growth of the AML cells through targeting miR-489-3p (Li et al., 2021). The transfer of miR-181 family to ALL cells has also been suggested to be associated with CNS involvement (Egyed et al., 2020). Moreover, it should be noted that not all the transfer of miRNAs are participated in the early stages of leukemogenesis and sometimes the transferred miRNAs are responsible of protecting the leukemic cells from the devastating effects of the anti-cancer agents. In this vein, it became evident that exosomes carrying miR-155 could confer drug-resistance against tyrosine kinase inhibitors in AML cells (Viola et al., 2016). The transfer of miR-19b and miR-20a has also been reported to be associated with induction of drug-resistance in APL cells through increasing the expression of multiple drug resistant proteins (MDPs) (Bouvy et al., 2017).

In addition to their key roles in the pathogenesis of acute leukemia, EVs are also a valid repertoire of genetic and epigenetic abnormalities of the parental cells. The next-generation sequencing (NGS) and GeneScan-based fragment-length analysis on the identified double-stranded DNA (dsDNA) in AML-derived EVs revealed that the majority of dsDNA mirror the mutations found in the genomic DNA obtained from primary leukemia cells (Kontopoulou et al., 2020; Bernardi et al., 2021). It has been suggested that there is a similar pattern between the expression of miRNAs in the EVs and pre-B ALL-derived leukemic cells (Xu et al., 2016). These findings together with the fact that these membrane bilayer-enclosed structures could be reachable in the body fluids suggest EVs as a valid tool for early detection, and for predicting patient outcome of both AML and ALL. The disease diagnosis/prognosis is not the only area that leukemia-derived EVs could be employed, as these groups of microvesicles could also integrate into the risk stratification of patients and even in the therapeutic strategies. To understand the clinical value of leukemia-derived EVs, this review aims to summarize the biology of EVs and discuss how EV-derived miRNAs could play a part in the development of AML and ALL. By combining the advances and challenges concerning the isolation of EVs, we discuss whether EVs could have a prognostic value in the clinical setting for leukemia patients.

## The Biology and Function of Extracellular Vesicles

### The Origin of Extracellular Vesicles

The evidence of the existence of EVs dated back to almost 60 years ago when the 20–50 nm-sized vesicles carrying clotting factors were identified in human platelet-free plasma (Wolf, 1967). In an article published by Wolf (1967), these lipid-rich vesicles were described as minute particulate material referred to as platelet dust because they were released from platelet (PLTs) during activation. The results of electronic microscopy revealed that these particles carry phospholipids and platelet factor 3 (tissue factor) to facilitate the coagulation process (Wolf, 1967). Since then, the definition and the perspective of this particle which is now named as EVs has changed. Now, EVs are envisioned as a group of lipid bilayer-enclosed structures that are released into the extracellular space (Doyle and Wang, 2019; Cocozza et al., 2020; Kalluri and LeBleu, 2020) by most cells to either discard their unwanted products or communicate with other neighboring or distant cells and even with extracellular matrix (ECM) (Doyle and Wang, 2019; Cocozza et al., 2020). This means that the content of EVs could be varied from proteins, lipids, and nucleic acids that could change the behavior of the recipient cells (Doyle and Wang, 2019; Cocozza et al., 2020). Thus far, several types of EVs have been identified in biological fluids such as urine, blood, ascites, and cerebrospinal fluid (Ibrahim and Marbán, 2016), and according to their size, these vesicles are classified into three groups: exosomes with the size range from 30 to150 nm, apoptotic bodies that have the size of 50–5,000 nm, and microvesicles (also known as ectosomes, shedding vesicles, or microparticles) with 100–1,000 nm in diameter (Kim et al., 2018; Liu et al., 2021). Apart from the size, the mechanism of release of EV differs from each other. While exosomes require multivesicular bodies (MVBs) for transportation, apoptotic bodies and microvesicles are released through interacting with the plasma membrane (Hessvik and Llorente, 2018). Among the mentioned EVs, it should be noted that exosomes have the most participation in intercellular communication, neuronal communication, antigen presentation, immune responses, organ development, and reproductive performance. This means that exosomes may have the most fundamental roles in pathophysiologic conditions, as well (Crenshaw et al., 2018; He et al., 2018). However, it should be noted that the size classification could not properly distinct microvesicles from exosomes, as not only both vesicles may overlap in the size but also both could be found in extracellular fluids and may exert similar intracellular effects (Zaborowski et al., 2015). Given these, in most cases, both microvesicles and exosomes are referred to as circulating vesicles (Raposo and Stoorvogel, 2013).

### Biogenesis of Circulating Vesicles

Like their intracellular functions, the biogenesis of circulating vesicles is complex and numerous internal and external factors are involved in this process. Different membrane receptors, lipid raft complexes, and endosomal sorting complexes regulate the biogenesis of EVs; however, this process could not be accomplished without the presence of Ras superfamily GTPase (Kim et al., 2018; Jadli et al., 2020).

#### Biogenesis of Exosomes

The biogenesis of exosomes includes three main steps; early endosomes (EEs) and late endosomes (LEs) or MVB formation, intraluminal vesicles (ILVs) formation, and the secretion of ILV into the extracellular space.

Early endosomes (EEs) form as a result of membrane budding inward the cell. The generated EEs subsequently fuse with endocytic vesicles to incorporate their cargos. After the evacuation of recycling endosomes from EEs, they undergo several stages of maturation to produce late endosomes (LEs), also known as multivesicular bodies (MVBs). There are two destinies for MVBs; either merge with the lysosome which leads to their destruction, or package their contents as 30–100 nm vesicles named intraluminal vesicles (ILVs) to integrate with the plasma membrane and release into the extracellular space (Akers et al., 2013). In this process, the companionship of several factors including cytoskeleton, motors proteins, and Rab family of GTPases may decide whether MVBs move toward lysosome or plasma membrane. For example, while ubiquitylated 7 guides MVBs toward lysosomal degradation, the members of the Rab family of GTPases such as Rab27A, Rab11, and Rab35 regulate the fusion of MVBs into the membrane through generating ILVs (Ostrowski et al., 2010).

The formation of ILVs consists of two main steps. At the primary stage, the endosome membrane should be organized by specific transmembrane proteins known as Tetraspanins (Pols and Klumperman, 2009). The presence of Tetraspanins is critical for the formation of ILVs, as these proteins construct Tetraspanin enriched microdomains (TEMs) domains within the membrane which could later cluster the essential proteins for ILVs generation (Hemler, 2005). Thus far, CD9 and CD63 are the most common as well as important Tetraspanins on the surface of ILVs that are also used as an identifier to isolate EVs in the body fluids (Jansen et al., 2009; Kosaka et al., 2010). The second step of ILVs formation is allocated to the addition of a group of multi-protein complexes named endosomal sorting complex required for transport (ESCRTs). The results of the in-depth molecular investigations showed that the presence of 4 types of ESCRT called ESCRT 0, I, II, and III is essential for membrane budding. Among them, the presence of ESCRT III is necessary for complete membrane budding. Upon EE formation and cargo mono-ubiquitination (Crenshaw et al., 2018; Mashouri et al., 2019), ESCRT-0 in the complex with hepatocyte growth factor-regulated tyrosine kinase substrate (HRS) and STAM1/2 recruit ESCRT I/II to bind to the phosphatidylinositol 3-phosphate (PIP3) located on the membrane. This event curves the membrane inward the lumen and also recruits the complex of ESCRT III-Alix- TSG101 that is essential for complete secretion of the vesicle from the membrane (Crenshaw et al., 2018; Juan and Furthauer, 2018; Mashouri et al., 2019; Jadli et al., 2020). It should be noted that if the cargo does not undergo mono-ubiquitination, ALG-2 interacting protein-X (ALIX) forms a team with Syntenin-Syndecan complex (Juan and Furthauer, 2018) or PAR1 (Kim et al., 2018; Skryabin et al., 2020) to mediate ESCRT-dependent secretion of exosomes (Kim et al., 2018; Mashouri et al., 2019).

Previous studies showed that even in the absence of HRS, Alix, and TSG101, the biogenesis of exosomes is continued. It has been suggested that this process could be mediated independently of ESCRT complex and through raft-dependent mechanisms. Sphingomyelin is a key player in this process (Skryabin et al., 2020), as its cleavage *via* sphingomyelinase 2 produces ceramide, a waxy lipid molecule which facilitates the formation of ILVs through regulating membrane budding (Juan and Furthauer, 2018). Moreover, ceramide could be catalyzed into sphingosine 1-phosphate (S1P) (Kajimoto et al., 2013), a signaling molecule which attracts cargos such as CD63, CD81, and flotillin into ILVs *via* interaction with inhibitory G protein (Gi)-coupled receptor. No matter through which mechanism ILVs may be produced within the MVBs. NSF attachment protein receptors (SNAREs) provide a platform for their secretion into the extracellular space. Through binding of Ca^2+^ to synaptotagmin VII, SNAREs complex could be activated, resulted in secretion of EVs (van Niel et al., 2018; Jadli et al., 2020). When the exosomes were released by the parental cells, VPS4 separates the remained components to recycle them for further uses.

#### Biogenesis of Micro Vesicles and Apoptotic Bodies

Since micro vesicles (MV) differ from the size from the exosomes, their biogenesis might have some differences with EVs. In the biogenesis of MV, actin rearrangement play a fundamental role (Gurunathan et al., 2021). In GTPase-dependent pathway, an enzyme named LIM kinase (LIMK) adds a phosphoryl group cofilin, which is a to actin depolymerizing enzyme, and thereby by inactivating this enzyme facilitate MV budding (Li B. et al., 2012). Another mechanism that could lead to MV biogenesis could be mediated through Small GTPase, ADP-ribosylation factor 6 (ARF6) signaling. Once ARF6 is activated, it could recruit ERK signaling pathway to activate myosin light-chain kinase (MLCK), which in turn activates myosin light-chain (MLC) at the necks of MVs. Activated MLCK interact with actin filaments, a process leads to MV production (Muralidharan-Chari et al., 2009). The biogenesis of MV could be triggered by phospholipid redistribution or cytoskeleton reorganization (Akers et al., 2013; D’Souza-Schorey and Schorey, 2018). The best example of a biological process that could activate MV biogenesis is the translocation of phosphatidylserine (PS) to the out membrane, a process that occur in the apoptosis (Akers et al., 2013; D’Souza-Schorey and Schorey, 2018). In apoptosis, cellular contents are packed in the form of small membrane-bound vesicles known as apoptotic bodies (ApoBDs), which contains externalized phosphatidylserine, calreticulin, and calnexin (Nunez et al., 2010). When actin-myosin located at the membrane starts to concentrate, a mechanism leads to membrane blebbing, the formation of ApoBDs begins (Xu et al., 2019). In addition to membrane blebbing, the reduction in the volume-to-surface ratio of cells also is another factor that provoke ApoBDs formation (Nunez et al., 2010).

### Extracellular Vesicles as a Vehicle to Deliver Essential Components to Target Cells

Based on the type of the parental cells and the mission that EV may have, the cargo of these circulating vesicles varies. However, proteins, lipids, metabolites, RNAs, and cDNAs are the common components of EVs (Crenshaw et al., 2018; He et al., 2018; Huang and Deng, 2019). Table 1 listed the most common molecules that could be detected in EVs, irrespective of their origin and cellular function.

**TABLE 1:** The content of circulating EVs.

**Types of cargo**	**Common contents**
***Proteins***	
Structural proteins	Tetraspanin proteins (CD81, CD82, CD37, and CD63), Syntenin, Alix, tumor susceptibility gene 101 protein (TSG101), Syndecans (SDC1–4), Intercellular adhesion molecule-1 (ICAM-1), Integrins, and Chaperons.
Outer membrane lipid-anchored proteins	CD39, CD73, CD55, CD59, Glypican-cellular prion protein (PrPC), and Amyloidogenic conformer.
Inner membrane lipid-anchored proteins	Small GTPases superfamily, and Protein kinases (Src).
Cell signaling proteins	Epidermal growth factor receptor (EGFR), Vascular endothelial growth factor receptor type-2 (VEGFR2), Insulin-like growth factor I receptor (IGF-1R), Notch receptors, Cytokine receptors, G protein-coupled receptors (GPCRs), Wnt proteins, Bone morphogenetic proteins (BMPs), Transforming growth factor β (TGF-β), tumor necrosis factor (TNF), TNF-related apoptosis-inducing ligand, first apoptosis signal (FAS) ligand, and extracellular matrix (ECM) proteins.
Enzymes	Phosphatases, Pyrophosphatases, Calcium-binding annexins, and phosphate transporters, RNA editing enzymes, Lipases, Proteases, Glycosyltransferases, Glycosidases, and Metabolic enzymes.
*LncRNAs*	lincRNA-p21, ANRASSF1, lncRNA SYSIL, lncRNA ROCK1, and lncRNA Paupar.
*miRNAs*	miR-150, -221, -1246, -140-3p, -16-5p, -20a-5p, -15a-5p, -17-5p, -18a, let-7b.

#### Proteins

Apart from structural proteins such as tetraspanins (CD81, CD82, CD37, and CD63), TSG101, Alix, and Rab family, heat shock protein, clathrin, protein kinase G (PKG), ATPase, syntenin, and RNA binding proteins (RBPs) are the most common types of proteins that are detected in EVs (Crenshaw et al., 2018; Huang and Deng, 2019; Jeppesen et al., 2019). It should be noted that the protein content of EVs depends on the original cell (He et al., 2018). MHC class II, ICAM-1, integrin, CD20, PD-L1, EGFR, IGF-1R, and cytokine receptors are among the most important proteins that are isolated from EVs (Pegtel and Gould, 2019).

#### Nucleic Acids

One of the most important contents of EVs is nucleic acids that could be either mitochondrial DNA (mtDNA), double strands DNA (dsDNA), single strands DNA (ssDNA) (Kalluri and LeBleu, 2020), or different types of RNAs ranging from coding RNAs such as mRNAs to non-coding species (Chu et al., 2020; Kalluri and LeBleu, 2020; Skryabin et al., 2020). Among different types of nucleic acids, it seems that microRNAs (miRNAs) are the most important cargos of EVs. Carolina Villarroya-Beltri and colleagues have successfully reveal that sumoylated hnRNPA2B1 and Ceramide are critical for loading miRNAs into EVs (Kosaka et al., 2010; Villarroya-Beltri et al., 2013). Upon integrating with mRNAs in the recipient cells, miRNAs could conveniently change their cellular behaviors. This process is well-defined in cancer cells, where the exosome miRNAs provide a platform for cancer cells to grow, invade into distant organs, and resist chemotherapeutic drugs (Ingenito et al., 2019; Rahbarghazi et al., 2019). Recently, it has been indicated that the resident-long non-coding RNAs in EVs could epigenetically alter the behavior of target cells (Wang et al., 2019; Da et al., 2021). The list of the most common miRNAs and lncRNA in EVs can be found in Table 1.

#### MicroRNAs

miRNAs are the best tools for regulating gene expression, either transcriptionally or post-transcriptionally (Huntzinger and Izaurralde, 2011; O’Brien et al., 2018). In complex with other proteins, which is called as MiRISC [miRNA and Argonaute 2(AGO-2)], the 5′-proximal region (nucleotide 2–8) of the miRNA binds to the 3′ UTR of the targeted mRNA and thereby inhibit or stimulate their expression. It should be noted that not all miRNAs bind to 3′ UTR of the mRNA and in some cases, miRNAs such as miR-10a could bind to 5′ UTR of the targeted mRNA (Ørom et al., 2008; Valinezhad Orang et al., 2014). Once miRNA interact to its targeted mRNA and if miRNA and miRNA response elements (MRE) are entirely complementary, the endonuclease activity of AGO-2 be provoked to cleave the mRNA (O’Brien et al., 2018). There are some piece of evidence suggesting that miRNAs could also enhance the expression of some genes (Huntzinger and Izaurralde, 2011). For example, when AGO makes a team with another protein associated to the miRNA-protein complex (microRNPs) named Fragile-x-mental, this complex could bind to AU-rich elements (AREs) at the 3′ UTR of the targeted mRNAs to enhance its expression. The best example of this regulatory process could be seen in Let-7 which by recruiting this mechanism could enhance the expression of the genes leading to cell cycle arrest (Forman et al., 2008). miR-24-1 is another miRNA that seems to could enhance the transcription of the target genes *via* inducing chromatin remodeling at enhancer site (Xiao et al., 2017). Given the importance of miRNAs in gene regulation, intense attention has been attracted to the biogenesis of these small non-coding RNAs.

#### Biogenesis of MicroRNAs

The biogenesis of miRNAs could be mediated through two main pathways; canonical and non-canonical mechanism (O’Brien et al., 2018). In canonical pathway, the primary miRNA (pri-miRNA) produced by RNA polymerase 2 converts to precursor-miRNA (pre-miRNA) by DROSHA/DiGeorge Syndrome Critical Region 8 (DGCR8) complex. The produced pre-miRNA, then, exported to the cytoplasm *via* exportin5, where it loses its pri-terminal miRNA’s loop *via* RNase III endonuclease Dicer. Helicase, then, come to play to convert the mature double stranded miRNA to single stranded miRNA (Han et al., 2004; Okada et al., 2009). In non-canonical pathway; however, the biogenesis of miRNAs could be mediated through Drosha/DGCR8 and Dicer-independent manner (O’Brien et al., 2018). In this manner, the non-cleaved pre-miRNA recruit exportin 1 to transport into the cytoplasm, where it makes a team with Argonaute-2 (AGO-2) to become mature (O’Brien et al., 2018).

#### Transfer of MicroRNAs to Extracellular Vesicles

Through binding to either RNA-Binding Proteins (RBP) or membrane proteins, miRNAs could be packaged into EVs (Groot and Lee, 2020). For example, RNA-Binding Proteins (RBP) such as heterogeneous nuclear ribonucleoprotein A2B1 (hnRNPA2B1) binds miRNA to transfer them into the EVs (Villarroya-Beltri et al., 2013). AGO-2, apart from its role in miRNA maturation, not only could aid miRNAs to transfer into EVs by recruiting the KRAS-MEK-ERK signaling pathway (Li L. et al., 2012; McKenzie et al., 2016) but also could protect them from degradation (Groot and Lee, 2020). Synaptotagmin-binding cytoplasmic RNA-interaction protein (SYNCRIP) is another protein that could join miRNA to transfer them into EVs. Through binding to extra-seed sequence (hEXO) motif of miRNAs, SYNCRIP aid miRNAs to gather into the exosomes (Santangelo et al., 2016).

### Extracellular Vesicles Transfer to Target Cells

As a carrier of information to the recipient cells, EVs should be delivered properly as well as safely to the target cells. Indeed, the vesicular structure of EVs protects their contents from enzymatic degradation and guarantees that the cargo be delivered to the target cells in its original state. One striking point about EV secretion is that under a stressful condition, cells are more prone to produce EVs (King et al., 2012). This phenomenon could be explained in two ways; first, this is an attempt of the cells to discard the damaging and harmful factors, or second, this is a communicational tool to aware the neighboring cells of the ongoing event. Valid evidence exists for both of these hypotheses. It has been reported that in response to the DNA damage and as the result of p53 activation, the pace of EVs release is exacerbated (Yu et al., 2006). Also, during hypoxia, the tendency of the cells to produce EVs is much higher than normoxic conditions (Park et al., 2010). No matter what is the purpose of EV secretion, when these circulating vesicles are released, they should be delivered to the recipient cells. The membrane-bound activating or inhibitory molecules in EVs transmit a signal to the recipient cells to allow EVs entrance. Like other circulating vesicles, EVs also integrates with the membrane of the responder cells *via* either endocytosis or membrane fusion. Once EVs enter the cells, they evacuate their valuable biologically active molecules repertoire to alter the cellular behavior. For example, the delivered miRNAs or lncRNAs could epigenetically change the expression of a wide range of molecules, activate/suppress different signaling pathways, and change the chromosomal structure. Also, the transferred mRNA could be translated into proteins that previously did not exist in the responder cells (Parolini et al., 2009).

## Extracellular Vesicles-Derived Micrornas, a Trojan Horse for Cancer Development

Although these seem to be striking methods to alter the characteristics of cells, production and delivery of EVs by cancer cells can not only transform the neighboring cells into the malignant counterpart but also engage the properties of the surrounding cells for their favor. The first evidence of the involvement of EVs in tumorigenesis was reported by Skog et al. (2008) who indicated that secreted EVs from glioblastoma cells enforce angiogenesis in brain endothelial cells. Very soon, the other pieces of evidence were found in other types of human cancers such as squamous cell carcinoma (Park et al., 2010), breast cancer (O’Brien et al., 2013; Zomer et al., 2015), and colorectal cancer (Tian et al., 2018), all suggesting that EVs act as Trojan horses to alter the microenvironment according to the needs of the tumor cells. Tumor-derived EVs could also induce immune exhaustion in the tumor microenvironment through upregulating the expression of inhibitory molecules of lymphocytes such as NK cells and CD8 positive T-lymphocytes or enhancing the differentiation of myeloid-derived suppressor cells (Lane et al., 2018). EVs also play a fundamental role in the pathogenesis of hematologic malignancies, as having a precise cross-talk with other residential cells within the BM niche is vital for the survival of hematologic malignant cells. The delivered EVs induce a “homing and nurturing” microenvironment in BM and protect the leukemic/neoplastic cells from the devastating effects of chemotherapeutic drugs. For example, it has been reported that chronic myeloid leukemia (CML)-derived EVs enforce BM stromal cells to produce IL-8, a cytokine which prolongs the survival of CML cells (Corrado et al., 2014). In multiple myeloma, it has been reported that the secreted EV from the BM mesenchymal stromal cells enhanced the proliferative capacity of multiple myeloma cells through transferring miR-15a (Roccaro et al., 2013). For acute type of leukemia (AML and ALL), there are multiple lines of evidence shedding light on the role of EVs in the pathogenesis of this common type of hematologic malignancy. In the following part of this article, we discuss the roles of EVs in the pathogenesis of acute leukemia.

## Extracellular Vesicles as a Communication Tool in Acute Leukemia

### Extracellular Vesicles Participate in the Pathogenesis of Acute Myeloid Leukemia and Acute Lymphoblastic Leukemia

In acute leukemia, EVs serve as a bridge to provide a dynamic cross-talk between leukemic cells and the stromal cells that reside in the BM niche. In another word, EVs are responsible for turning BM microenvironment into a leukemia-permissive space. There are multiple evidence suggest that EVs might have a key role in the early stages of leukemogenesis. It has been revealed that the transfer of EVs from the leukemic cells to either HSCs or myeloid progenitor cells could abrogate the proper differentiation and thereby lead to the development of leukemia. Moreover, the transferred EVs protect leukemic cells from apoptotic stimuli such as chemotherapeutic drugs (Kumar et al., 2018). In the following part of the paper, more details will be discussed about the participation of EVs in the pathogenesis of leukemia.

#### The Role of Extracellular Vesicles Derived MicroRNAs in the Regulation of Leukemogenesis

**FIGURE 1:**
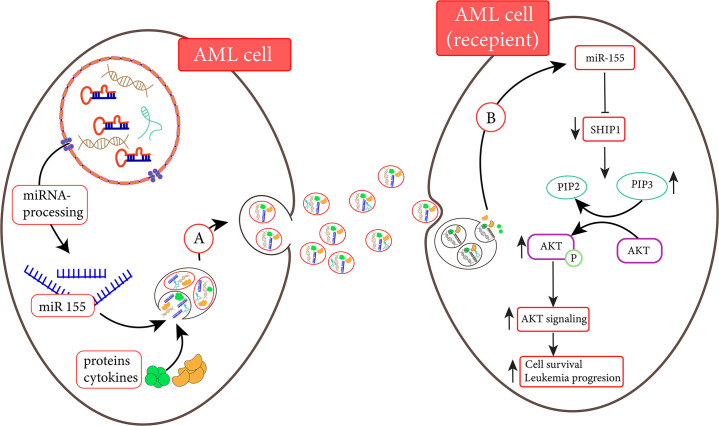
AML cells are able to transfer miR-155 to another AML cell (Hornick et al., 2015). (A) MiR-155 and other elements are transferred to recipient cells *via* exosomes. (B) MiR-155 derived from exosomes is released into the recipient cell and inhibits the activated SH2 domain-containing inositol 5′-phosphatase1 (SHIP1) expression. Following the inhibition of SHIP1, the enhancement of AKT signaling promotes cell survival (Xue et al., 2014).

One of the main mechanisms through which EVs promote the progression of leukemia is mediated through delivering the essential oncogenic RNAs into HSCs to change its characterization for developing into leukemic cells. In this process, the delivery of miRNAs plays fundamental roles. In ALL, for instance, it has been claimed that the leukemic cells released EVs containing miR-43a-5p to the BM microenvironment. After internalizing to the BMSCs, this miRNA targets Wnt signaling axis and thereby inhibits osteogenesis in the BM. The malignant HSCs transform to leukemic cells as osteogenesis is suppressed (Yuan et al., 2021). In addition, it has been found that the secreted EVs from BMSCs consisting of miR-21 are delivered into HSCs and consequently enhance the development of B-ALL cells. On the other hand, the exo-miR-21 could interact with TGF-β and shut down the anti-tumor immune responses in the BM microenvironment (Lv et al., 2021). The same mechanism was also observed in AML. A previous study showed that the serum level of EVs carrying miR-10b is elevated in AML patients as compared to the healthy counterparts (Fang et al., 2020). miR-10b is notorious for its role in halting the granulocytic/monocytic differentiation in HSCs and enhancing the proliferative capacity of immature myeloid progenitors, leading to AML development (Bi et al., 2018). The large amount of EVs containing miR-10b in newly diagnosed AML patients suggested the significant roles of this delivering system in inducing AML (Fang et al., 2020). MiR-4532 is another delivered miRNA that could be transferred to HSCs through AML-derived EVs to suppress the expression of LDOC1. LDOC1 is an inhibitor of the STAT-3 signaling pathway and thereby its downregulation leads to STAT-3 activation. When AML-derived EVs deliver such cargo to the HSCs, they manipulate the proliferative capacity of these cells by stimulating the STAT-3 signaling pathway (Zhao et al., 2019). The results of the previous studies also indicated that some of the leukemic-derived EVs could induce early leukemogenesis in myeloid progenitors through transferring miR-155 (Figure 1). Through binding to 3/UTR of c-Myb, miR-155 inhibits the expression of this differentiating transcription factor in myeloid cells and thereby induces differentiation arrest, a critical step in AML development (Hornick et al., 2016). Apart from miRNAs, some evidence suggest the exosomal transfer of oncogenic mRNAs such as those encoding NPM1 and FLT3-ITD to the myeloid progenitors from leukemic cells could also lead to leukemia development (Huan et al., 2013). By silencing the expression of hematopoiesis-related growth factors such as IGF-1, CXCL12, KIT ligand, and IL-7, AML-derived EVs could enforce neoplastic HSCs committed to the myeloid progenitor, enhancing the production of leukemic cells (Kumar et al., 2018). The transfer of the anti-apoptotic proteins such as MCL-1, BCL-2, and BCL-XL to the immature myeloid blasts could also guarantee their survival in the BM microenvironment (Wojtuszkiewicz et al., 2016). In ALL, it has been found that leukemia-derived EVs could induce metabolic switch in BMSCs. Johnson et al. (2016) have proposed that the leukemia-derived EV recipient stromal cells have minimal mitochondrial aspiration and use an aerobic glycolysis instead of oxidative phosphorylation, which in turn could provide the desired energy for ALL development in the BM microenvironment. It seems that during leukemogenesis, EVs act as a Trojan horse by delivering either miRNAs or onco-mRNAs. These tiny vesicles could alter the characteristic of BMSCs in a way that they increase the possibility of AML or ALL development.

#### The Role of Extracellular Vesicles Derived MicroRNA in Regulating Survival of Leukemic Cells

As mentioned earlier, one of the main purposes of EVs delivering in acute leukemia is to evolve a leukemia-permissive space in BM, where leukemic cells could have an opportunity to survive, proliferate and grow. The number of studies that cover this mechanism in the progression of both AML and ALL is skyrocketed over the last decades and thus far, numerous molecules have been identified to be involved in this process. EVs could potentiate the survival and proliferative potential of ALL. In a study conducted by Patel et al. (2016), it has been reported that when non-proliferating ALL cells were cultured with ph^+^ ALL-derived exosomes, their proliferative capacity were vigorously reinforced, suggesting that the contents of these EVs might have proliferative factors. But what components could be involved in this process? Haque and Vaiselbuh (2020) came up with the answer when they successfully isolated miR-181 from the EVs in the serum of pediatric cases of ALL. By conducting further analysis, they proposed that the delivered miR-181, on one hand could enhance the expression of anti-apoptotic proteins such as MCL-1 and BCL-2 in leukemic cells, and on the other hand, could elevate the expression of proliferation-related genes, including PCNA and Ki-67. Moreover, the authors also claimed that the up-regulation of miR-181 in EVs-derived from ALL patients suppressed the expression of pro-apoptotic genes. As a straightforward interpretation of these results, it was proposed that ALL cells might have longer survival and more potent proliferative capacity by uptaking these EVs (Haque and Vaiselbuh, 2020).

**FIGURE 2:**
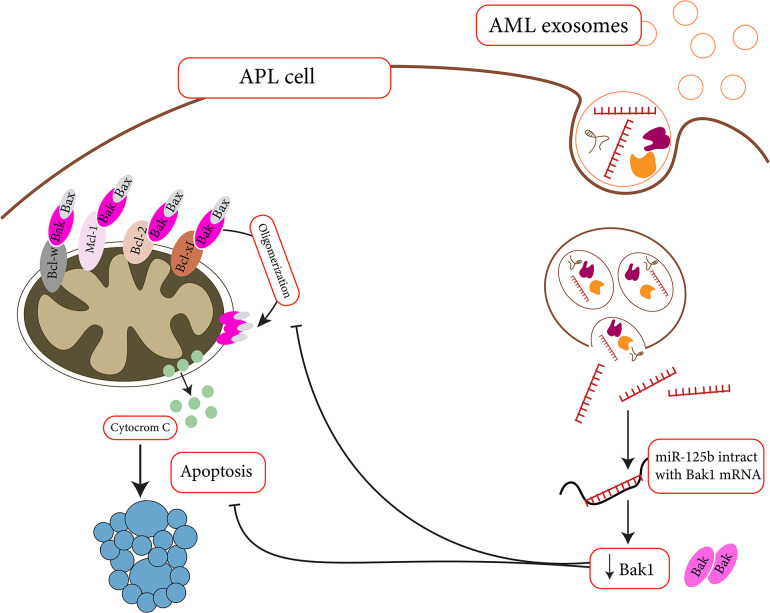
Apoptosis suppression by miR-125b might help APL cells survive in cytotoxic situation (Zhang et al., 2011). MiR-125b is a microRNA that targets Bak1. As miR-125 levels rise, Bak levels fall, and apoptosis decreases.

For AML, multiple studies declared the importance of EVs in increasing the survival of leukemic cells. Through delivering DKK1 to the BM stromal cells (BMSCs), for example, AML-derived EVs could halt the progression of hematopoiesis and osteoblast differentiation in the BM niche and promote the uncontrolled proliferation of leukemic cells (Kumar et al., 2018). BMP-2 is another cargo that could be transmitted between AML cells and the mesenchymal stem cells (MSCs) in the BM microenvironment to guarantee the survival of leukemic cells. The excessive amount of BMP-2 in leukemic cells transfer in the form of EVs to MSCs, where this transcription factor could reinforce osteogenic differentiation. As a result, through secretion of connective tissue growth factor (CTGF) from MSCs, AML cells could find a chance to grow (Battula et al., 2017). So far, many exosome-delivered miRNAs have been identified for endowing the AML cells the survival advantages. MiR-125b, for instance, is one of these delivered miRNAs that target the expression of pro-apoptotic proteins such as BAK and Bmf, and P53 in AML cells (Figure 2). This miRNA could not only halt the induction of apoptosis in AML cells but also induce cell proliferation through promoting cell cycle (Bousquet et al., 2010; Zhang et al., 2011; Vargas Romero et al., 2015). Ji et al. (2021) have also suggested that BMSC-derived EVs could enhance AML development through delivering miR-26a-5p to leukemic cells. By activating the Wnt/B-catenin signaling pathway, the delivered miR-26a-5p promotes AML cell proliferation, migration, and invasion (Ji et al., 2021). Not all the alterations should be delivered by exo-miRNAs and in some cases exo-lncRNAs might also have a role in the regulation of the BM microenvironment. Exo-circ-0009910 has been claimed to block the expression of miR-5195-3p and thereby enhance the progression of cell cycle in AML cells through up-regulating the growth factor receptor-bound protein 10 (GRB10) (Wang et al., 2021). Another exo-lncRNA that has been detected in the sera of AML patients is Circ-0004136, which is a sponge for miR-570-3p, a tumor suppressor miRNA that reduced the expression of TSPAN3 in AML cells. When EVs containing Circ-0004136 is delivered into AML cells, not only the viability of the cells be sustained but also these cells proliferate more autonomously (Bi et al., 2021).

#### The Role of Extracellular Vesicles in Regulating Angiogenesis

Since the density of BM microvessels is one of the main criteria that could protect the survival of leukemic cells in the BM, it comes as no surprise that EVs might participate in the regulation of the density of BM microvesicles. Through delivering angiogenic factors/proteins and miRNAs, EVs change the characteristics of endothelial cells and promote angiogenesis (Huan et al., 2013). The angiogenic contents of EVs increase the proliferative capacity of endothelial cells, enforce their invasion and subsequently enhance the expression of proangiogenic factors such as IL6 and VEGF. Fang et al. (2016) reported that the transferred EVs could deliver IL-8 and VEGF to endothelial cells to change their angiogenic signature and thereby prolong the survival of NB4 cells. Among the different angiogenic cargos, perhaps the miR-17-192 family is the most important non-coding RNAs that could stimulate angiogenesis in acute leukemia (Doebele et al., 2010). Through suppressing integrin A5 in human umbilical vein endothelial cells (HUVECs), it has been proposed that miR-92a, one of the members of the miR-17-92 family, could increase the density of microvesicles in the BM microenvironment (Xin et al., 2017). Apart from direct delivery of angiogenic factors, leukemic cells could also induce hypoxia in endothelial cells through EVs carrying HIF-1α. In response to hypoxia, endothelial cells activate their angiogenic properties, so that the new vessels might bring enough supplies of oxygen to the cells (Park et al., 2010). In ALL cases, the delivered exo-miR-181 could also be transferred into endothelial cells and enforce these cells to produce VEGF to enhance angiogenesis (Patel et al., 2016). Overall, these findings shed light on the importance of EVs in the progression and dissemination of myeloid leukemia cells through regulating the angiogenic process.

#### The Role of Extracellular Vesicles in Regulating Drug-Resistance

Constructing a leukemia-friendly environment, EVs also protect leukemic cells from anti-cancer agents. Viola et al. (2016) was the first group who have reported that the transferred TGF-β and miR-155 from AML cells to MSCs provide a chemotherapy-protective environment for AML cells. Wojtuszkiewicz et al. (2016) realized that the chemo-sensitive AML cells could acquire the resistant phenotype through receiving Bcl-2 containing EVs from the chemo-resistant AML cells. They proposed that EVs are communicational tools for inducing drug resistance (Wojtuszkiewicz et al., 2016). The same results were also reported by Bouvy et al. (2017) who indicated that daunorubicin-resistant AML cells could induce drug resistance in other leukemic cells through delivering drug efflux pump multidrug resistance protein 1 (MRP-1). Aberrant delivery of the anti-apoptotic molecules/proteins to AML cells is well-studied in numerous studies and has been suggested as a mechanism to increase the survival of leukemic cells against anti-cancer agents. For example, miR-155 and miR-375 enriched EVs could be transferred from BMSCs to confer drug resistant phenotype in AML cells against tyrosine kinase inhibitors (Viola et al., 2016). MiR-19b and miR-20 could induce chemo-resistance through activating TGF-β and PI3K/Akt signaling axis (Tazzari et al., 2007). In the induction of resistance against immune-therapies, the footprint of EVs is also observed. Hong et al. (2017) suggested that AML-derived EVs could attenuate the efficacy of adoptive natural killer (NK) cell therapy by delivering inhibitory ligands that counteract the activity of NKG2D receptor on NK-92 cells. The transferred TGF-β from AML cells into NK-92 cells reduced the expression of NKG2D in these cells through recruiting TGFβRI/II pathway (Hong et al., 2017).

**FIGURE 3:**
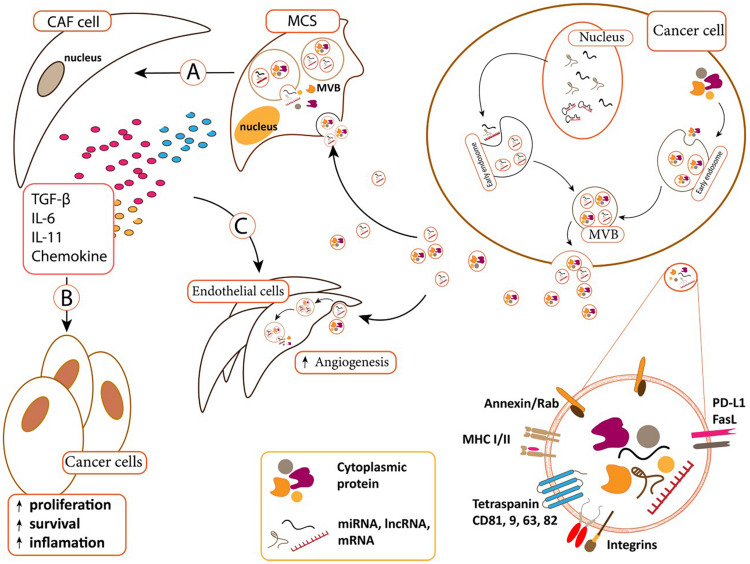
(A) Cancer-derived exosomes can transfer to MSCs and change MSCs. MSCs differentiated to cancer-associated fibroblast (CAF) in B.M microenvironment. (B) CAFs supports cancer cells *via* secreted content such as TGF-β, IL-11, IL-6, and chemokine. CAF secreted contents affect cancer cells and increase cancer cell proliferation, survival. (C) Cancer derived exosomes can also uptake by endothelial cells and induce angiogenesis. CAFs secreted content can also induce angiogenesis in endothelial cells (Padua and Massagué, 2009; Mineo et al., 2012; Pickup et al., 2013; Costanza et al., 2017).

The number of studies demonstrated the role of exo-miRNAs/proteins in conferring drug resistant phenotype is rare in ALL cases. It has been reported that when BMSCs absorb leukemia-derived EVs, they would be transformed into cancer associated fibroblasts (CAFs) that could evolve a protective niche against chemotherapeutic drugs (Figure 3). On the reciprocal manner, BMSCs-derived EVs could deliver galectin-3 into ALL cells to increase their drug resistance through activating NF-κB signaling axis (Fei et al., 2015).

### Extracellular Vesicles and Acute Leukemia Prognosis

Given the importance of EVs in the pathogenesis of acute leukemia, many studies have come to a consensus that analyzing the contents of these circulating vesicles could provide a valuable perspective about the outcome of patient with leukemia. Table 2 summarized the results of several studies evaluating the prognostic value of EVs in leukemia patients.

**TABLE 2:** The correlation between leukemia-derived EVs and the outcome of patients.

**Study**	**Results**	**References**
**AML**		
Bernardi et al.	AML patients with higher exosome levels of miR-10b had shorter survival as compared to those with lower levels of miR-10b.	Bernardi and Farina, 2021
Bernardi et al.	The higher expression of exosome miR-532 in AML patients is associated with lower survival, suggesting that miR-532 can act as an independent prognostic marker in AML.	Bernardi and Farina, 2021
Fang et al.	The higher expression of miR-10b in exosomes harvested from the sera of AML patients is associated with the aggressive clinical characteristics of the disease and poorer outcomes in the patients.	Fang et al., 2020
Jiang et al.	The identification of miR-125b in EVs of AML patients is suggestive of elevated risk of disease relapse and shorter 2-years overall survival, introducing miR-125b as an independent prognostic marker.	Jiang et al., 2018
Kontopoulou et al.	Genetic analysis of EVs harvested from pediatric AML patients could be used as a tool to evaluate MRD in the patients.	Kontopoulou et al., 2020
Chen et al.	EVs encapsulating miR-1246 could increase the survival of leukemic stem cells (LSCs) in AML patients through targeting LRIG1 and STAT-3 signaling pathway and thereby induce poor outcomes in the patients.	Chen et al., 2021
Bouvy et al.	MRP-1 proteins could be delivered from chemo-resistance HL-60 cells to chemo-sensitive leukemic cells. Circulating EVs containing miR-19b and miR-20a are responsible for the induction of chemo-resistance in AML patients and thereby reduce their overall survival.	Bouvy et al., 2017
Barzegar et al.	AML-derived EVs containing MRD proteins could induce chemo-resistance against idarubicin, suggestive of the participation of EVs in the induction of poor prognosis in AML patients.	Barzegar et al., 2021
Viola et al.	BMSC-derived EVs that contain miR-155 could confer drug resistance against tyrosine kinase inhibitors in AML patients, reduce the opportunity of complete remission in patients.	Viola et al., 2016
Lin et al.	Elevated plasma exosome-derived miR-532 is associated with favorable outcomes in AML patients.	Lin et al., 2020
Jiang et al.	An increased in the expression of exosome miR-125b could increase the risk of relapse in AML patients and is associated with the reduced overall survival.	Jiang et al., 2018
Hong et al.	The reduction in the levels of plasma EV-TGFβ1 protein in AML patients who received chemotherapy is indicative of the favorable response to treatment and induction of long-term complete remission. Changes in EV-TGFβ1 levels in AML patients could be considered a prognostic and risk stratifying factor.	Hong et al., 2017
Hornick et al.	Elevated serum EV levels containing let-7a, miR-99b, -146a, and -191, is associated with poor prognosis in AML patients.	Hornick et al., 2015
Caviano et al.	EV-derived miR-155 is a well-known independent prognostic factor for AML patients. The level of this miRNA correlates with the number of WBCs and complex karyotypes in patients.	Caivano et al., 2017
**ALL**		
Egyed et al.	The elevation in EV containing miR-181a could be an indicator for CNS involvement for the pediatric patients with ALL.	Egyed et al., 2020
Labib et al.	The upregulation of extracellular miR-22 in pediatric ALL is associated with poor prognosis and shorter overall survival.	Labib et al., 2017
Rzepiel et al.	Detetction of miR-128-3p and miR-222-3p in blood of ALL patients could be indicator of MRD and thus far these circulating miRNAs could be considered a prognostic maker for ALL patients.	Rzepiel et al., 2019

## Challenges of Extracellular Vesicles in the Clinical Application for Acute Leukemia

Given the importance of EVs in the pathogenesis of leukemia and based on the number of reports suggesting the benefits of evaluating EVs contents for early diagnosis or predicting the outcome of patients, it could be known that EVs are promising circulating biomarkers. However, the story is not as simple as it looks. The study of EVs as biomarkers in clinical approaches is still a new field, and no standard methods have been established yet for the proper enrichment and isolation of these circulating vesicles. The diversity in the protocols used for EVs isolation, enrichment, and measurement leads to the fact that in many cases, the results of studies are not comparable with each other, and this may have a negative effect on the validation of the results (Lane et al., 2018). For example, for EVs isolation, based on the available equipment and materials, methods such as differential ultracentrifugation, density gradient ultracentrifugation, polymer-facilitated precipitation, immune-affinity isolation and, size exclusion chromatography (SEC) are used. Among them, differential ultracentrifugation is considered a gold standard method for EV isolation (Théry et al., 2006). Despite the great popularity, ultracentrifugation could lead to vesicle aggregation and contamination of the protein contents of EVs (Baranyai et al., 2015; Nordin et al., 2015). Moreover, the variability in the methods in evaluating pelleting efficiency has led to the discrepancy in results obtained from different studies. To tackle the contamination problem, density gradient ultracentrifugation was developed (Kalra et al., 2013; Choi and Gho, 2015). However, the difficulty in the procedure of this method and the risk of loss of samples have muted the enthusiasm in employing density gradient ultracentrifugation (Muller et al., 2014). For the polymer-facilitated precipitation and the immune-affinity isolation, the poor purity of isolated EVs and the lack of proper antibodies have been claimed as the main factors that could restrict the efficacy of the methods (Van Deun et al., 2014; Lane et al., 2018).

The diversity in the isolation techniques could not only produce variability in the results but also influence the precise molecular characterization of EVs. The high purity of EVs and ability to distinct the EV-derived proteins/nucleic acids from non-EV sources are essential for the proper characterization of EVs. Apart from this, thus far, several techniques, including transmission electron microscopy (TEM), dynamic light scattering (DLS), nanoparticle tracking analysis (NTA), flow cytometry, and tunable resistive pulse sensing (TRPS) are used for the physical characterization of EVs. Although effective, each of these techniques also have some limitations and complications. Problems such as vesicle shrinkage and the limited number of vesicles that could be analyzed by TEM have made flow cytometry superior to TEM which is currently a gold standard method (Van der Pol et al., 2014; Erdbrügger and Lannigan, 2016). However, the application of flow cytometry is not without practical limitation, as the small size of EVs and their low refractive index result in improper scattering of the vesicles by flow cytometry (Van Der Pol et al., 2012; Nolan, 2015). The labeling of EVs with protein-dye was not successful in tackling this problem, as EVs possess a restricted amount of target molecules (Welsh et al., 2017). A similar problem could be seen in studies using DLS and NTA for the molecular characterization of EVs. Although these techniques are rapid, i.e., NTA could measure the contents of thousands of single EVs in less than a few minutes (Bi et al., 2021) and could analyze bulk samples, only larger particles with the ability to scatter the greater amount of light signal could be characterized by these methods (Anderson et al., 2013; Lane et al., 2018). The nature of TRPS is different from other methods and this non-optical measurement technique uses the electrical impedance for analyzing the physical characterization of EVs. However, the necessity of an expert user to operate this technique has made it difficult to use TRPS for the characterization of EVs (Lane et al., 2018). It should be noted that both qRT-PCR and Western blotting analysis for evaluating the miRNA/RNA and protein content of EVs could also be affected by the methods that are used for the isolation of EVs. Taken together, all mentioned limitations suggest the necessity of an appropriate protocol for EV handling and measurement. The more accurate the methods, the faster EVs could be employed in clinical settings for risk stratifying patients.

## Can Extracellular Vesicles Meet the Clinical Challenges for Risk Stratification in Patients With Acute Leukemia?

The application of EVs in risk stratification for patients with acute leukemia is beneficial, though it has a long way to be achievable. First, as mentioned earlier, due to the limitation of all mentioned isolation techniques in distinguishing exosomes from micro-vesicles, both of these cellular components referred to as circulating vesicles, which limits our understanding of the property of these circular vesicles. The less we know about the contents of circulating EVs, the longer it might take for receiving approval EVs as a diagnostic tool in clinical application (Witwer et al., 2013). Moreover, some studies revealed that the level of circulating EVs could be affected by numerous factors, and thereby, EVs varied based on the time of sample collection (Pickup et al., 2013). This finding threatens the value of EVs in the clinical application and prioritizes the importance of an optimized protocol for the collection, isolation, and storage of EVs. Additionally, many studies have thus far evaluated the efficacy and the value of EVs in *in vitro* analysis. So, experiments conducting on patient’s samples are required to achieve better results and provide a wider perspective about the application of EVs as a prognostic factor in patients with acute leukemia.

## Conclusion

From the first description of EVs in the samples of patients with leukemia, there is no doubt that these tiny circulating vesicles play fundamental roles in leukemogenesis, cell proliferation, survival, and also angiogenesis. The components of EVs conveniently alter the structure of BM in the way that it protects leukemic cells from either the adaptive arm of the immune system or anti-cancer agents. Thus far, many studies are focusing on the importance and value of EVs in determining the outcome of patients with leukemia; however, a long way should be passed to reach the best results. As it was mentioned earlier, according to the size of EVs, three classes of these circulating vesicles have been identified in body fluids and for sure, each of them may participate in some specific biological processes. However, due to the size overlap and the disability of the current isolation and characterization technologies, it is impossible to distinguish these vesicles from each other and analyze their cargos individually. Moreover, many of the results published in this area are conflicting and incomparable, as each research group might use different techniques for the collection, processing, and storage of EVs. Given these limitations, it seems that more issues should be addressed before EVs could enter into the clinical application for acute leukemia. Nevertheless, the journey of EVs in leukemia is still mesmerizing.

## Author Contributions

MI: conceptualization, literature survey, figure designing, review structure, writing review and editing, and references collection. ZH: conceptualization, literature survey, review structure, writing review and editing, and references collection. FJ: literature survey, writing review, and references collection. AH and AG: critical review and editing. Y-DL: formating and reference collection. LJ: conceptualization, literature survey, writing review and editing, references collection, tables preparation, supervision, and correspondence. Z-SC: critical review and editing, correspondence, and supervision. All authors contributed to the article and approved the submitted version.

## Conflict of Interest

The authors declare that the research was conducted in the absence of any commercial or financial relationships that could be construed as a potential conflict of interest.

## Publisher’s Note

All claims expressed in this article are solely those of the authors and do not necessarily represent those of their affiliated organizations, or those of the publisher, the editors and the reviewers. Any product that may be evaluated in this article, or claim that may be made by its manufacturer, is not guaranteed or endorsed by the publisher.
